# Burden stratification and spatial analysis for malaria chemoprevention in Malawi

**DOI:** 10.5281/zenodo.22207863

**Published:** 2026-08-31

**Authors:** Chisomo Mkwandah, Alinafe Maenje, Steven Munharo, Michael Msukuma, Donnie Mategula

**Affiliations:** 1 Infectious Diseases and Epidemiology Research Group, Malawi Liverpool Wellcome Programme, Queen Elizabeth Central Hospital Campus, Chipatala Avenue, P.O. Box 30096, Chichiri, Blantyre 3, Malawi.; 2 Department of Agricultural and Applied Economics, Lilongwe University of Agriculture and Natural Resources, Bunda College Campus, P.O. Box 219, Lilongwe, Malawi.; 3 The School of Global and Public Health, Kamuzu University of Health Sciences, Private Bag 360, Blantyre, Malawi.; 4 Department of Clinical Sciences and International Public Health, Liverpool School of Tropical Medicine, Liverpool, L3 5QA, United Kingdom.

## Abstract

**Background:**

Malaria remains a leading cause of morbidity and mortality in Malawi, particularly among children under five years of age, with substantial geographic variation in transmission. Although malaria chemoprevention is effective, evidence on how malaria burden, climatic conditions, population exposure, and spatial dependence can inform its geographic prioritisation in Malawi remains limited. This study aimed to develop composite measures of malaria burden, climatic suitability, and population exposure, assess climatic influences and spatial dependence on malaria transmission and identify geographic priority areas for malaria chemoprevention.

**Methods:**

District-month malaria surveillance, climate, and population data from 2021–2023 were analysed. Composite indices and spatial mapping assessed malaria burden, climatic suitability, and population exposure. Fixed-effects and spatial panel models assessed climatic influences and spatial dependence. These indicators were integrated into a composite priority score to identify geographic priorities for chemoprevention.

**Results:**

Nkhata Bay, Nkhotakota, Salima, and Neno were classified as very high-burden districts, while Mwanza was classified as high burden. Rainfall was positively associated with malaria incidence, whereas rainfall anomalies were negatively associated with transmission (both p < 0.001). Significant spatial dependence (ρ = 0.34–0.49; p < 0.001) indicated geographic interdependence in malaria outcomes. Integrated analysis identified priority areas, particularly in central and southern Malawi, where epidemiological, climatic, population, and spatial risks converged.

**Conclusions:**

Integrating malaria burden, climatic suitability, population exposure, and spatial dependence can support evidence-based geographic prioritisation of malaria chemoprevention and targeted malaria control planning in Malawi.

## Introduction

Malaria remains one of the leading global public health challenges. According to the World Malaria Report 2025, an estimated 282 million malaria cases and more than 600,000 malaria-related deaths occurred worldwide in 2024 [1], with most of the burden concentrated in the World Health Organization (WHO) African Region. Despite substantial progress in malaria prevention and control over the past two decades, sustained transmission continues across much of sub-Saharan Africa because of favourable ecological conditions, climatic variability, socioeconomic vulnerability, and inequities in access to effective malaria interventions [2,3].

Malaria chemoprevention has become an increasingly important component of malaria control strategies. By administering full therapeutic courses of antimalarial medicines to individuals at risk, malaria chemoprevention can substantially reduce malaria morbidity and mortality among vulnerable populations [4,5]. Seasonal Malaria Chemoprevention (SMC) has consistently reduced clinical malaria episodes by more than 60% among eligible children in the Sahel and is recommended by the WHO for areas with highly seasonal malaria transmission [5–7]. Earlier evidence from intermittent preventive treatment also demonstrated substantial reductions in malaria morbidity and mortality among children and pregnant women [8]. More recently, WHO expanded its recommendations to include Perennial Malaria Chemoprevention (PMC) for areas with perennial malaria transmission, recognising that chemoprevention strategies should be tailored to local epidemiological settings [4].

Malawi experiences perennial malaria transmission with seasonal peaks during the rainy season between November and April. Despite considerable investments in insecticide-treated nets (ITNs), indoor residual spraying (IRS), prompt diagnosis and treatment, intermittent preventive treatment during pregnancy (IPTp), and integrated vector management, malaria remains endemic and continues to impose a substantial health burden, particularly among children under five years of age.

According to the World Malaria Report 2025, Malawi recorded approximately 6.38 million malaria cases in 2024, ranking among the countries with the highest malaria burden globally [1]. National surveillance data further indicate that transmission is disproportionately concentrated in southern and lakeshore districts, where favourable climatic conditions, extensive mosquito breeding habitats, and the predominance of *Plasmodium falciparum* sustain high transmission [9,10]. These patterns are increasingly influenced by climate variability and extreme weather events, contributing to substantial temporal and spatial heterogeneity in malaria transmission.

Malaria transmission in Malawi is highly heterogeneous across districts. Differences in rainfall, temperature, altitude, ecological conditions, population density, housing quality, poverty, healthcare access, and demographic characteristics result in marked variation in malaria risk and transmission intensity [9,11,12]. Consequently, districts experience markedly different epidemiological conditions despite implementing broadly similar malaria control interventions, including ITNs, focal IRS, case management, and IPTp [13,14]. Such heterogeneity limits the effectiveness of geographically uniform intervention strategies and allows persistent transmission hotspots to remain.

Although malaria chemoprevention has not yet been adopted as a national intervention for children in Malawi, several clinical studies have generated evidence supporting its potential use in specific populations. Early school-based research demonstrated that screening and treating primary school children reduced *P. falciparum* infection and anaemia, although the authors suggested that repeated treatment or more sustained preventive approaches could achieve greater health benefits [15].

More recently, an open-label randomised controlled trial in Machinga District compared intermittent preventive treatment with intermittent screening and treatment for school-based malaria control, providing direct evidence on alternative malaria prevention strategies among Malawian schoolchildren [16]. The Chemoprevention of Malaria in Children with Sickle Cell Anaemia (CHEMCHA) programme further evaluated malaria chemoprevention among children with sickle cell anaemia in Malawi and Uganda, with the protocol comparing dihydroartemisinin–piperaquine and sulfadoxine–pyrimethamine [17], and the completed trial demonstrating that weekly dihydroartemisinin–piperaquine was more effective than monthly sulfadoxine–pyrimethamine in preventing malaria in this high-risk population [18]. In addition, the ongoing CHAMP Drug Trial is evaluating multiple antimalarial drugs, administered individually and in different combinations, to identify the most effective and safe chemoprevention regimens for young children in Malawi and generate evidence to inform future malaria chemoprevention policies in Malawi and other countries in eastern and southern Africa [19,20]. Together, these studies demonstrate growing national and regional interest in malaria chemoprevention and provide important evidence on the effectiveness of different chemoprevention strategies. However, they do not identify which geographic areas should be prioritised if malaria chemoprevention is introduced more broadly in Malawi

Although previous studies have described spatial variation in malaria burden and investigated individual climatic, environmental, and socioeconomic determinants of malaria transmission [2,20,21], few have integrated epidemiological, climatic, demographic, and spatial dependence into a single decision-support framework for prioritising malaria chemoprevention. Most studies focus on describing malaria distribution rather than identifying where preventive interventions should be targeted. Consequently, there remains limited evidence to support geographically and demographically targeted implementation of malaria chemoprevention in Malawi's heterogeneous transmission settings.

To address this gap, this study integrated district-level malaria surveillance, climate, and population data from 2021 to 2023 to identify geographic priorities for malaria chemoprevention in Malawi. Specifically, the study sought to: i. Develop composite measures of malaria burden, climatic suitability, and population exposure across districts in Malawi; ii. Assess the influence of climatic factors and spatial dependence on malaria transmission using fixed-effects and spatial panel econometric models; iii. Identify geographic priority areas for malaria chemoprevention by integrating epidemiological, climatic, population, and spatial risk indicators.

## Methods

The study was conducted in Malawi, a south-eastern African country, which is divided into 28 districts. Each district has a district hospital that reports routine malaria surveillance data to the National Malaria Control Programme (NMCP), except Mzimba, which has two (Figure 1). These 28 districts served as the spatial units of analysis. Malawi has a subtropical climate with a unimodal rainy season from November to April and a dry season from May to October. Its ecological zones range from cooler high-altitude plateaus to warmer low-lying lakeshore areas and the Lower Shire Valley. These environmental gradients drive substantial variation in rainfall, temperature, and mosquito breeding habitats, resulting in pronounced spatial heterogeneity in malaria transmission. Although malaria is endemic nationwide, the burden is highest in hot, humid lowland districts along Lake Malawi and the Lower Shire Valley, with relatively lower transmission in some highland districts. Given the predominantly rural population and variable access to health services, district-level routine data from hospital catchment areas provide a suitable basis for identifying priority areas for chemoprevention.

**Figure 1.**
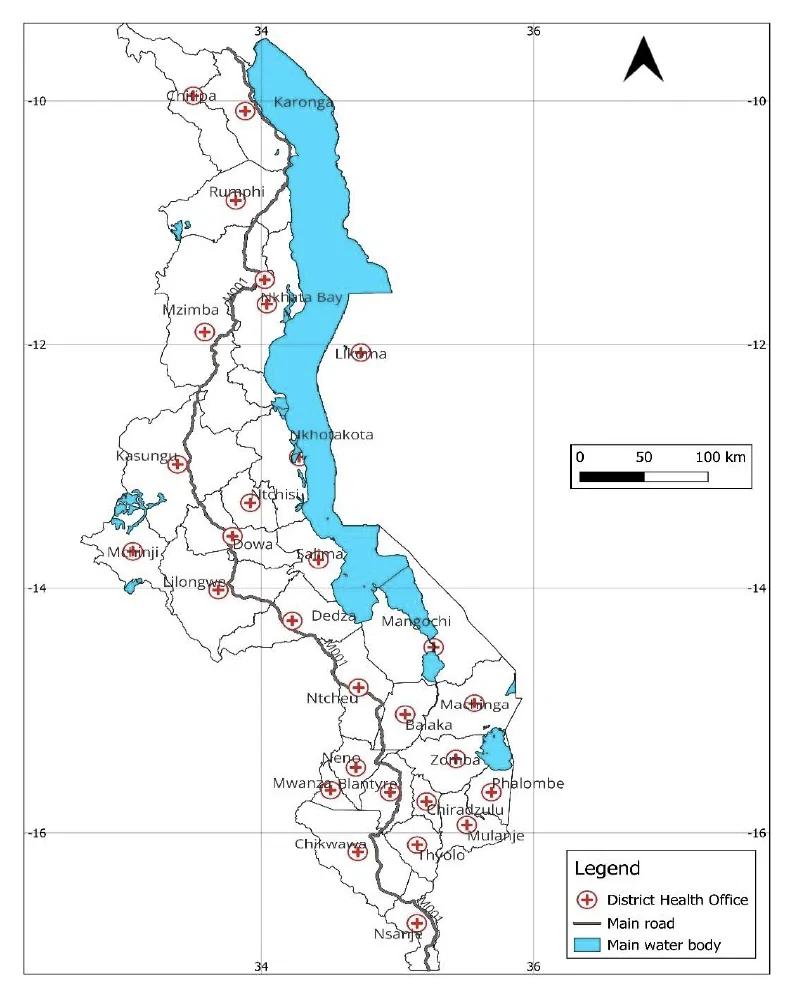
Geographic distribution of district hospitals included in the study across Malawi.

Data for this study were obtained from routine national malaria surveillance and climate monitoring systems. Malaria case and mortality data were sourced from the NMCP monthly reporting system, which compiles outpatient and inpatient confirmed malaria cases, under-five cases and malaria-attributed deaths from all district hospitals and associated health facilities. Climate information, including monthly rainfall, rainfall anomalies and temperature measures, was obtained from a harmonised climate and environmental monitoring dataset derived from national meteorological records and satellite observations. Population denominators for total and under-five populations were drawn from the NMCP routine reporting platform. All datasets were cleaned, harmonised by district and month and integrated into a single district-month panel covering the period from 2021 to 2023. These combined routine data sources formed the basis for the spatial and temporal analyses.

### Theoretical framework

This study was informed by two complementary frameworks: the Ross–Macdonald malaria transmission theory [22–24] and spatial dependence theory [25–27]. Together, these frameworks provide the theoretical foundation for examining how climatic, demographic, environmental, and geographic factors influence malaria transmission and generate spatial dependence in malaria burden across districts in Malawi.

The Ross–Macdonald framework remains the central epidemiological basis for understanding malaria transmission. It describes how mosquito biology, human susceptibility and environmental conditions jointly determine transmission intensity [28]. The theory emphasises the role of rainfall, humidity and temperature in shaping mosquito density, parasite development and survival. These relationships are captured by the classical reproduction number:

$R_{0}=\frac{ma^{2}bce^{-\mu \tau }}{ru}$

where *m* is the mosquito-to-human ratio (mosquito density per human), *a* is the mosquito biting rate, *b* is the probability of transmission from an infectious mosquito to a susceptible human, *c* is the probability of transmission from an infectious human to a susceptible mosquito, *μ* is the mosquito mortality rate, *τ* is the parasite's extrinsic incubation period, and *r* is the human recovery rate. Recent studies show that climatic anomalies and temperature shifts can significantly alter transmission patterns across African settings [28]. In Malawi, hotter low-land districts such as Nsanje, Chikwawa, Salima and Mangochi consistently exhibit high transmission, reflecting the mechanisms described in this model. Climatic and demographic variables used in this study therefore operationalise the pathways through which the Ross–Macdonald theory predicts variations in malaria burden.

Spatial dependence theory explains that malaria outcomes in one district are influenced by conditions in neighbouring districts through shared ecological environments, human mobility and vector movement [29,30]. Recent work has demonstrated strong spatial clustering of malaria incidence and vector behaviour across Sub-Saharan Africa, showing that neighbouring districts often share similar transmission profiles due to these interlinked processes [31]. This dependence is formally represented in spatial econometric models. The spatial lag formulation captures endogenous spillover effects:

$Y_{it}=\rho WY_{it}+X_{it}\beta +\alpha_{i}+\varepsilon_{it}$

where *Y_it_* denotes the malaria outcome in district *i* at time *t*, *W* is the spatial weights matrix, *ρ* is the spatial autoregressive coefficient measuring spillover effects from neighbouring districts, *X_it_* is the matrix of explanatory variables, *β* is the vector of regression coefficients, *α_i_* represents district fixed effects, and *ε_it_* is the idiosyncratic error term. The spatial error formulation accounts for spatial correlation in unobserved influences:

$Y_{it}=X_{it}\beta +\alpha_{i}+\xi_{it}$

where the spatial error process is defined as

$\xi_{it}=\lambda W\xi_{it}+\varepsilon_{it}$

Where λ measures the degree of spatial dependence in unobserved ecological or structural factors, W is the spatial weights matrix, and ε_it_ is a random error term assumed to be independently and identically distributed. Evidence from recent spatial analyses shows that failing to account for these dependencies can produce biased estimates and understate the true influence of climate and environmental drivers [31]. In Malawi, districts that lie close to one another often share similar climatic conditions, settlement patterns and health system challenges, leading to spatial clustering of high malaria transmission, particularly in the Southern region and lakeshore belt. Recognising these spatial linkages is therefore essential for accurate modelling and for identifying priority districts for chemoprevention.

### Model description

We applied a combination of non-spatial and spatial panel modelling approaches to examine how climatic variability and demographic structure influence malaria outcomes across Malawi's districts. Monthly district-level observations were assembled into a panel dataset covering the study period, allowing both cross-sectional differences between districts and temporal variation within districts to be analysed simultaneously. The modelling strategy follows the classical formulation of panel econometric models [32], together with established spatial econometric extensions [25–27]. These frameworks allow malaria outcomes in one district to depend on conditions in neighbouring districts through spatial lag processes, while also accounting for spatially correlated unobserved factors through spatial error processes.

To characterise district-level malaria intensity, malaria incidence among children under five years was calculated as the number of confirmed malaria cases per 1,000 under-five population. The under-five incidence indicator for district *i* is defined as:

$I_{i}=\frac{C_{i}}{P_{i}^{u5}}\times 1000$

where *C_i_* denotes the total number of confirmed malaria cases among children under five in district i, and *P_i_^u5^* denotes the total under-five population in the same district.

Because malaria burden is multidimensional, three complementary indicators were used to capture disease intensity: total malaria incidence per 1,000 population, under-five incidence per 1,000 under-five population, and total malaria deaths. Since these indicators are measured on different scales, they were standardised using the z-score transformation [33]:

$Z\left(x_{i}\right)=\frac{x_{i}-\bar{x}}{s_{x}}$

where *x_i_* represents the value of indicator *x* in district *i, x̄* denotes the mean of the indicator across all districts, and *s_x_* represents the corresponding standard deviation.

A composite malaria burden profile was then constructed as a weighted combination of the standardised indicators:

$B_{i}=0.50Z\left({\text{Inc}}_{i}\right)+0.30Z\left({\text{U5Inc}}_{i}\right)+0.20Z\left({\text{Deaths}}_{i}\right)$

where Inc_i_ is total malaria incidence per 1,000 population, U5Inc_i_ is under-five malaria incidence per 1,000 under-five population, and Deaths_i_ represents the total malaria deaths recorded in district *i*. All indicators were standardised using z-scores to ensure comparability across variables measured on different scales before aggregation into a composite burden index. Composite malaria indices combining multiple epidemiological indicators have been widely used in malaria stratification and spatial risk assessment to capture both transmission intensity and health outcomes across geographic areas [3,14,34,35]. Higher values of *B_i_* therefore indicate districts with relatively elevated malaria transmission and mortality burden compared with the national distribution. To facilitate comparability across indicators and across years, the burden score was transformed to a unit interval using global min-max normalisation:

$B_{i}^{*}=\frac{B_{i}-\min\left(B\right)}{\max\left(B\right)-\min\left(B\right)}$

where min(*B*) and max(*B*) denote the minimum and maximum burden scores observed across all districts and years. Malaria burden stratification was based on a composite burden score 36,35,3. The stratification demarcation is shown below in Table 1.

**Table 1:** Malaria burden stratification based on the composite malaria burden score.

Burden Tier	Burden Score Threshold
Very high burden	≥ 0.70
High burden	0.50-0.69
Moderate burden	0.25-0.49
Lower burden	< 0.25

Climatic receptivity to malaria transmission was captured through a climate risk index derived from rainfall and temperature anomalies. For each district *i*, the climate risk score was defined as the average of the z-standardised absolute anomalies in rainfall and temperature variables:

$CI_{i}=\frac{Z\left(|R_{i}|\right)+Z\left(|T_{mean,i}|\right)+Z\left(|T_{max,i}|\right)+Z\left(|T_{min,i}|\right)}{4}$

where *R_i_* denotes rainfall anomaly, *T_mean,i_* denotes mean temperature anomaly, *T_max,i_* denotes maximum temperature anomaly, and *T_min,i_* denotes minimum temperature anomaly. Taking the absolute value captures the magnitude of climatic deviations irrespective of direction, reflecting conditions that may enhance malaria transmission potential [37].

The climate risk index was subsequently transformed to a comparable scale between zero and one using min-max scaling:

$CI_{i}^{*}=\frac{CI_{i}-\min\left(CI\right)}{\max\left(CI\right)-\min\left(CI\right)}$

Human exposure to malaria risk was represented by demographic structure and population concentration. For each district, three demographic indicators were considered: total population *P_i_^all^*, under-five population *P_i_^u5^*, and the proportion of children under five within the total population. The under-five population share was defined as:

$S_{i}=\frac{P_{i}^{u5}}{P_{i}^{all}}$

These indicators were standardised and aggregated into a human exposure index defined as:

$H_{i}=0.50Z\left(P_{i}^{u5}\right)+0.30Z\left(S_{i}\right)+0.20Z\left(P_{i}^{all}\right)$

where greater weight is assigned to the under-five population because young children represent the demographic group most vulnerable to malaria infection and mortality.

The human exposure score was then transformed to a comparable scale between zero and one:

$H_{i}^{*}=\frac{H_{i}-\min\left(H\right)}{\max\left(H\right)-\min\left(H\right)}$

Finally, the equal-weight approach was adopted because the three dimensions represent complementary determinants of malaria risk, and no robust empirical evidence exists to justify differential weighting. This provides a transparent and reproducible framework for district prioritisation, consistent with established approaches to composite indicator development [33,38]:

${\text{CompositePriorityScore}}_{i}=\frac{CI_{i}^{*}+H_{i}^{*}+B_{i}^{*}}{3}$

This equal-weight aggregation approach reflects the absence of strong empirical evidence to justify differential weighting across these dimensions and is consistent with established practices in malaria risk stratification and spatial prioritisation frameworks [33]. The resulting composite index provides a relative ranking of districts according to overall malaria risk and supports the identification of priority areas for targeted malaria interventions.

Peak malaria transmission periods were determined using monthly district-level malaria case. For each district *i*, rolling five-month cumulative case totals were calculated across all consecutive five-month windows. The window with the maximum cumulative number of cases was identified as the peak transmission period.

Formally, the peak period for district *i* is defined as:

${\text{PeakPeriod}}_{i}=\arg\max_{t=1,\mathrm{dots},8}\mathrm{left}\left(\sum_{m=t}^{t+4}C_{i,m}\mathrm{right}\right)$

where *C_i,m_* denotes the number of malaria cases reported in district i during month *m*.

This data-driven approach identifies the consecutive five-month window with the highest malaria burden, enabling the determination of district-specific transmission peaks over the study period [11,7]. It is consistent with established practices for empirically defining seasonal high-burden intervals from routine surveillance data and aligns with WHO guidance on targeting interventions during periods of concentrated transmission, typically occurring within short consecutive windows of approximately three to five months [1].

### Econometric methods

Non-spatial fixed-effects panel models were estimated to control for unobserved, time-invariant district characteristics that may be correlated with climatic conditions. For district *i* at time *t*, the baseline specification is:

$Y_{it}=\alpha_{i}+\beta^{T}C_{it}+\gamma Pop_{it}+\varepsilon_{it}$

where *Y_it_* denotes a district-level malaria outcome, *C_it_* is a vector of climatic covariates, *Pop_it_* is total population size, *α_i_* captures district fixed effects, and *ε_it_* is the idiosyncratic error term. Estimation uses the within (fixed-effects) transformation, exploiting within-district variation over time while absorbing time-invariant district heterogeneity [32,39].

Because climatic variables can be highly correlated (e.g., mean/max/min temperature and rainfall level vs anomaly measures), model specifications were assessed for collinearity and interpretability. The preferred specification includes rainfall level, mean temperature, and population size, while alternative specifications substitute rainfall anomalies and/or temperature extremes as robustness checks. Malaria outcomes were modelled as continuous district-level indicators using linear fixed-effects regression, which is appropriate for monthly aggregated rates and large population denominators. Hausman tests were used to compare fixed and random effects; fixed effects were retained to ensure consistency and comparability across outcomes [32].

Malaria transmission is inherently spatial due to mosquito dispersal, human mobility, shared ecological environments, and correlated climate patterns across neighbouring districts. Empirical evidence consistently shows strong spatial clustering in malaria risk [3,2,12]. Ignoring spatial dependence can lead to biased or inefficient estimates and underestimated uncertainty [25,26]. To account for spatial dependence, spatial panel models [27] were estimated on a balanced panel subset to ensure a consistent district–time structure for spatial estimation.

A *k*-nearest neighbour (kNN) spatial weights matrix with *k* = 3 was constructed using district centroids. The kNN approach was chosen because it guarantees a connected spatial structure despite heterogeneity in district size and shape, while limiting the inclusion of distant neighbours that may not share similar malaria transmission dynamics. The weights were row-standardised so that spatial lags represent weighted averages of neighbouring districts [27,40]. Spatial panel models were then estimated using the same covariates as the non-spatial models while accounting for spatial spillovers (SAR) and spatially correlated unobserved factors (SEM). The spatial lag (SAR) model is:

$Y_{it}=\rho \sum_{j}w_{ij}Y_{jt}+\alpha_{i}+X_{it}\beta +u_{it}$

where *∑_j_w_ij_Y_jt_* is the spatially lagged outcome capturing exposure to neighbouring malaria levels, *ρ* measures the strength of spatial spillovers, *α_i_* denotes district fixed effects, *X_it_* includes climate and population covariates, and *u_it_* is an idiosyncratic disturbance. This specification captures diffusion-like processes in which malaria outcomes in one district are influenced by outcomes in neighbouring districts [25, 26].

The spatial error (SEM) model is:

$Y_{it}=\alpha_{i}+X_{it}\beta +\xi_{it}$

with the spatial error process:

$\xi_{it}=\lambda \sum_{j}w_{ij}\xi_{jt}+\varepsilon_{it}$

where *ξ_it_* is an error component that is spatially correlated across districts, *λ* measures the degree of spatial autocorrelation in unobserved shocks, and *ε_it_* is a spatially uncorrelated idiosyncratic error term. The SEM formulation captures spatial clustering arising from omitted ecological or structural factors that vary across space but are not fully observed [26,27].

### Model validation

Model validation involved several diagnostic tests to assess model specification and spatial dependence. Hausman tests indicated that fixed effects were preferred over random effects for most malaria indicators, confirming the need to control for unobserved district-level characteristics [32]. Moran’s I tests applied to the residuals of the non-spatial fixed effects models revealed significant positive spatial autocorrelation (p < 0.001), indicating clustering of malaria outcomes across neighbouring districts. Robust Lagrange Multiplier tests further confirmed the presence of both spatial lag and spatial error dependence, supporting the use of spatial panel models [25,27]. Consistent with these diagnostics, the spatial lag models produced positive and significant spatial autoregressive parameters (*ρ*), while the spatial error models produced significant spatial error parameters (*λ*), indicating that malaria outcomes are influenced both by neighbouring districts and by spatially correlated unobserved factors. Variance Inflation Factors indicated no problematic multicollinearity among climate variables, and robust standard errors were used to account for heteroskedasticity. Overall, these diagnostic results indicate that the spatial fixed effects models provide a reliable representation of district-level malaria patterns.

### Ethical considerations

This study relied entirely on secondary, anonymised data and was therefore exempt from ethical review. No personal identifiers or precise household locations were included, preventing any form of re-identification. The permission to use the malaria surveillance dataset was secured from the NMCP through its formal data request procedures.

## Results

### Descriptive spatial patterns

Figure 2 shows that maximum temperature across Malawi remained highest in the southern areas while relatively cooler conditions were observed in the northern and high-altitude areas. Rainfall patterns were more variable, with higher rainfall mainly in the northern and southern districts in 2021–2022 but expanding into parts of the central region in 2023.

**Figure 2.**
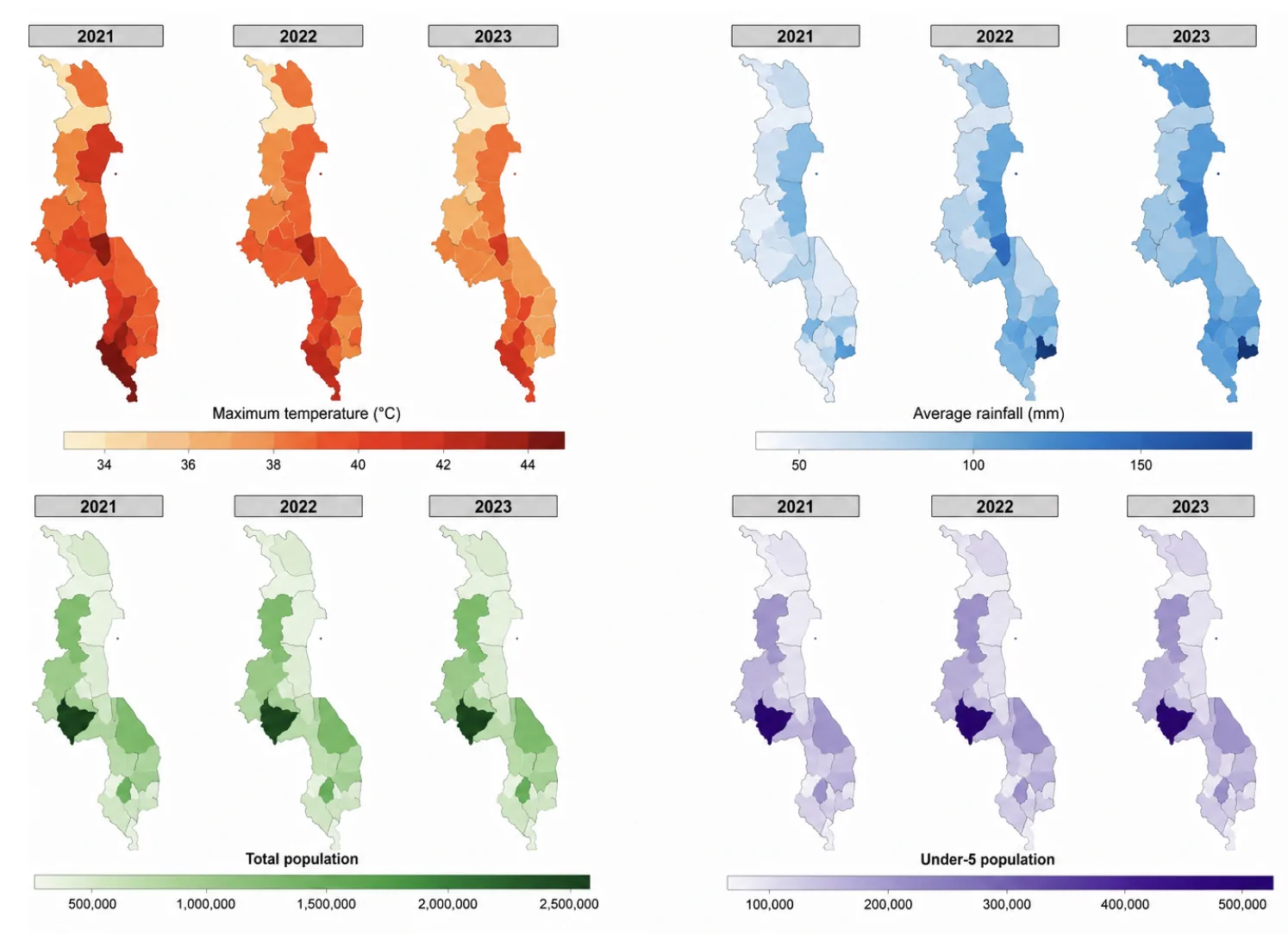
District-level spatial distribution of maximum temperature, average rainfall, total population, and under-five population across Malawi, 2021–2023.

Figure 2 also shows a clear spatial concentration of population in the major urban districts. Lilongwe, Mzuzu, and Blantyre consistently recorded the highest levels of both the total and under-five population.

Malaria transmission intensity showed geographic clustering across districts (Figure 3). Higher transmission levels were concentrated in southern districts and lakeshore areas, while northern highland districts showed comparatively lower transmission intensity, especially in 2022. Central districts exhibited moderate transmission levels.

**Figure 3.**
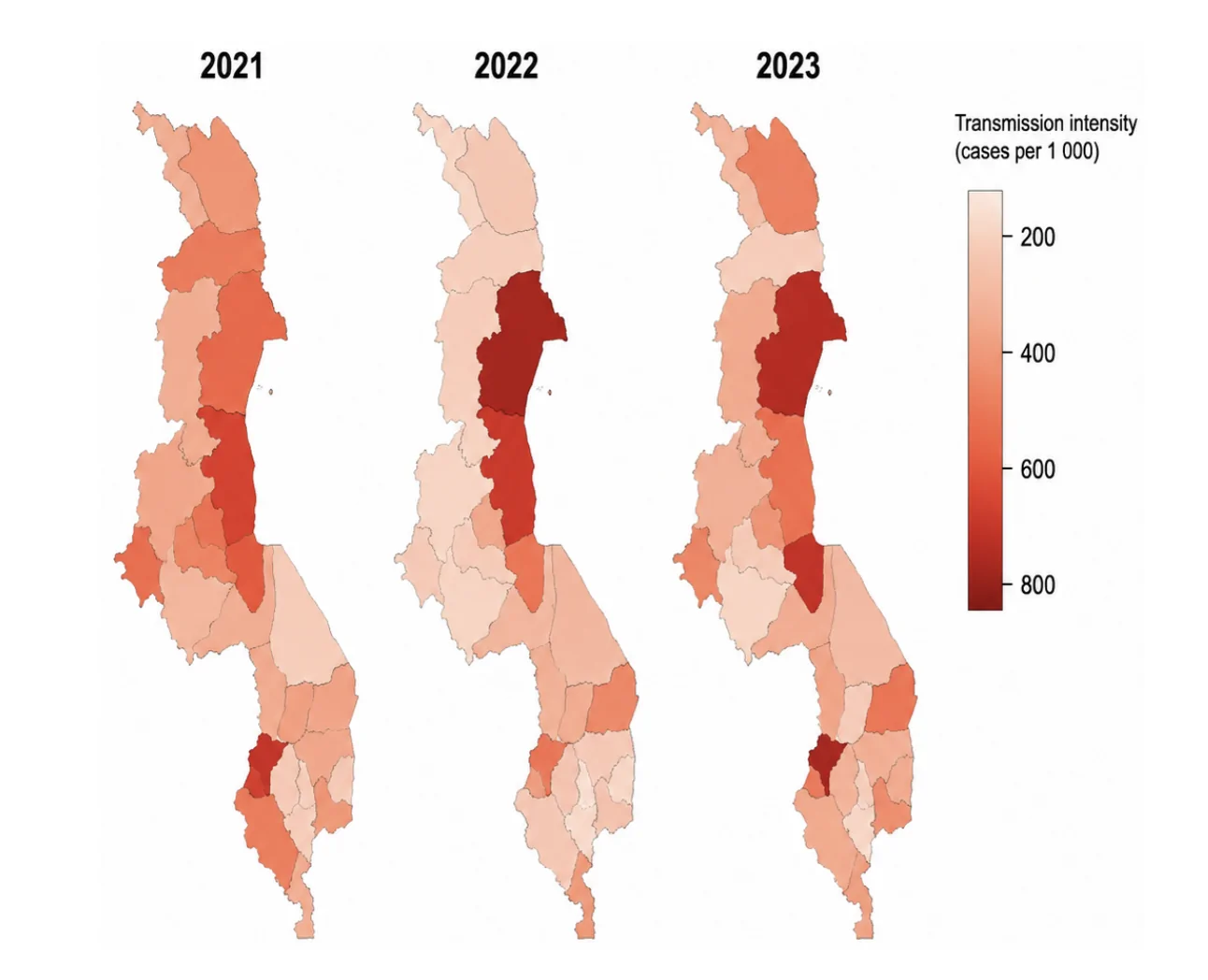
Spatial distribution of malaria transmission intensity across districts in Malawi, 2021–2023.

Figure 4 illustrates the spatial distribution of malaria transmission intensity across Malawi during the dry (May–October) and wet (November–April) seasons. Transmission intensity was consistently higher during the wet season, with several districts in the central and southern regions exhibiting persistently elevated case rates.

**Figure 4.**
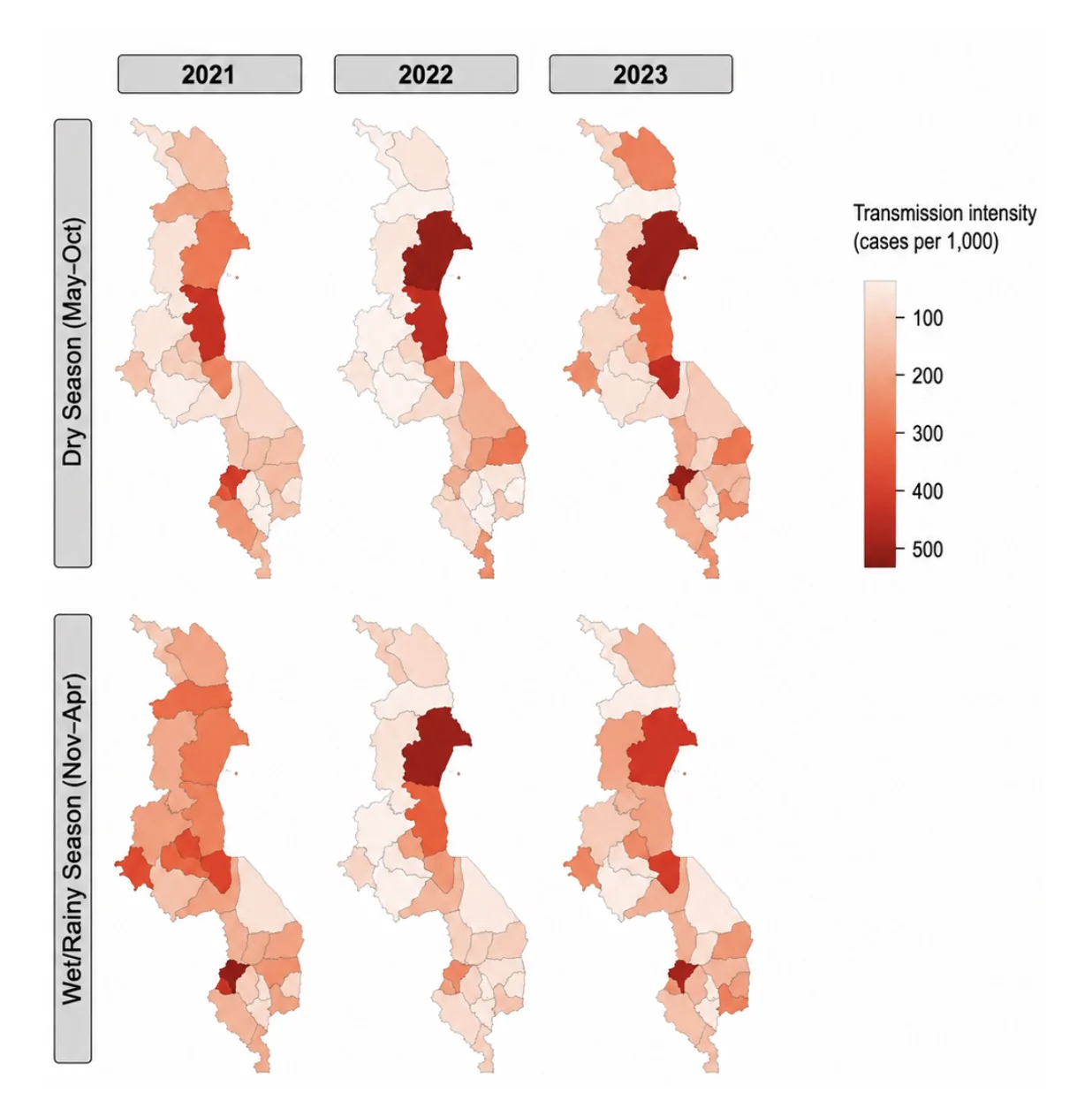
Seasonal distribution of malaria transmission intensity across districts in Malawi during the dry and wet seasons, 2021–2023.

The spatial distribution of climate risk across Malawi revealed notable regional variation, with higher risk levels concentrated in several southern districts and comparatively lower levels observed in the northern and parts of the central regions (Figure 5). This pattern highlights the greater climate-related vulnerability faced by districts in the southern region relative to other parts of the country.

**Figure 5.**
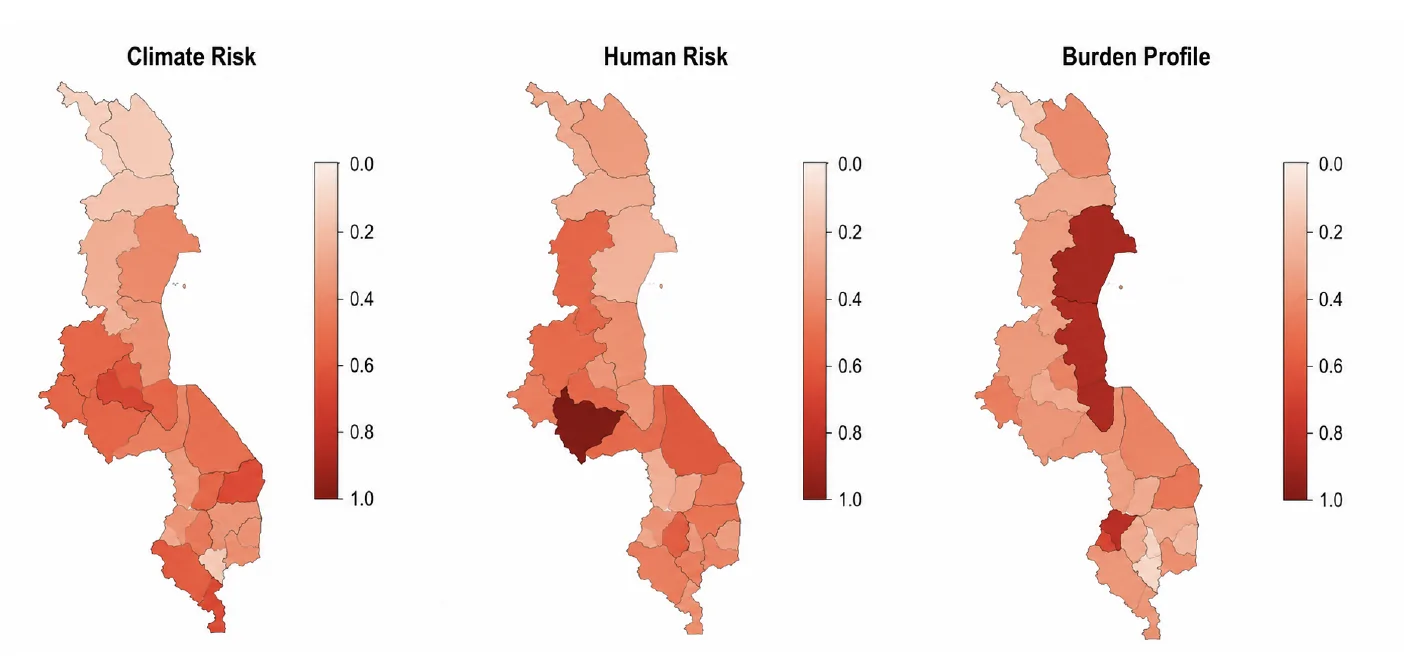
Spatial distribution of climate risk, human risk, and malaria burden profiles across districts in Malawi, 2021-2023.

Human risk also exhibited clear spatial variation across Malawi, with elevated levels observed in several central and southern districts, while relatively lower levels were evident in the northern region (Figure 5). The highest concentrations appeared in parts of the central region, indicating areas where human-related vulnerability factors are most pronounced.

The composite malaria burden index (Figure 5) shows higher burden levels in central and southern districts, while northern districts fall within lower burden categories, as shown in Table 2.

**Table 2:** Classification of districts by composite malaria burden profile.

Burden Tier	Districts
Very high burden (≥ 0.70)	Nkhata Bay, Nkhotakota, Salima, Neno
High burden (0.50 – 0.69)	Mwanza
Moderate burden (0.25 – 0.49)	Machinga, Mchinji, Ntchisi, Mangochi, Mulanje, Dedza, Lilongwe, Likoma, Ntcheu, Kasungu, Karonga, Chikwawa, Nsanje, Mzimba, Balaka, Dowa, Zomba
Lower burden (< 0.25)	Rumphi, Blantyre, Phalombe, Chitipa, Thyolo, Chiradzulu

Seasonal malaria transmission patterns vary across districts in Malawi. As shown in Table 3, the most common peak transmission period occurs between March and July, covering eight districts. This is followed by January to May and August to December, each covering six districts. Other districts have different peak periods, including February to June, April to August, May to September, June to October, and July to November, showing that the timing of malaria transmission is not the same across the country.

**Table 3:** Seasonality patterns by peak malaria transmission period across districts in Malawi.

Peak Transmission Period	Districts
January – May	Neno, Mwanza, Dedza, Lilongwe, Rumphi, Thyolo
February – June	Karonga
March – July	Salima, Ntcheu, Chikwawa, Blantyre, Kasungu, Balaka, Chitipa, Chiradzulu
April – August	Phalombe
May – September	Nsanje
June – October	Machinga, Likoma, Mangochi
July – November	Nkhata Bay, Nkhotakota
August – December	Mulanje, Mchinji, Ntchisi, Zomba, Mzimba, Dowa

These differences show that malaria seasonality varies a lot across districts in Malawi. This means that interventions should not follow a single national schedule but should instead be timed to match local transmission patterns. This is especially important for planning interventions such as seasonal malaria chemoprevention, which need to be delivered during periods when malaria risk is highest.

### Malaria chemoprevention priority

The classification of districts into priority strata, based on the composite index identifies marked variation in malaria-related risk when jointly considering population vulnerability, climatic conditions, and disease burden (Figure 6). Lilongwe, Nkhata Bay, Nkhotakota, and Salima are classified among the highest-priority districts, reflecting consistently elevated levels across these underlying dimensions. Additional districts identified as high priority include Neno, Mwanza, Dowa, Kasungu, Mchinji, Mangochi, and Mulanje, indicating that elevated risk is observed across multiple regions of the country. Moderate-priority districts constitute a substantial proportion of districts, suggesting intermediate levels across the composite indicators, whereas low-priority districts are relatively fewer and correspond to comparatively lower combined risk. Collectively, these results demonstrate clear heterogeneity in priority levels across districts and underscore the importance of adopting differentiated, evidence-based planning approaches informed by the composite index, rather than uniform national strategies.

**Figure 6.**
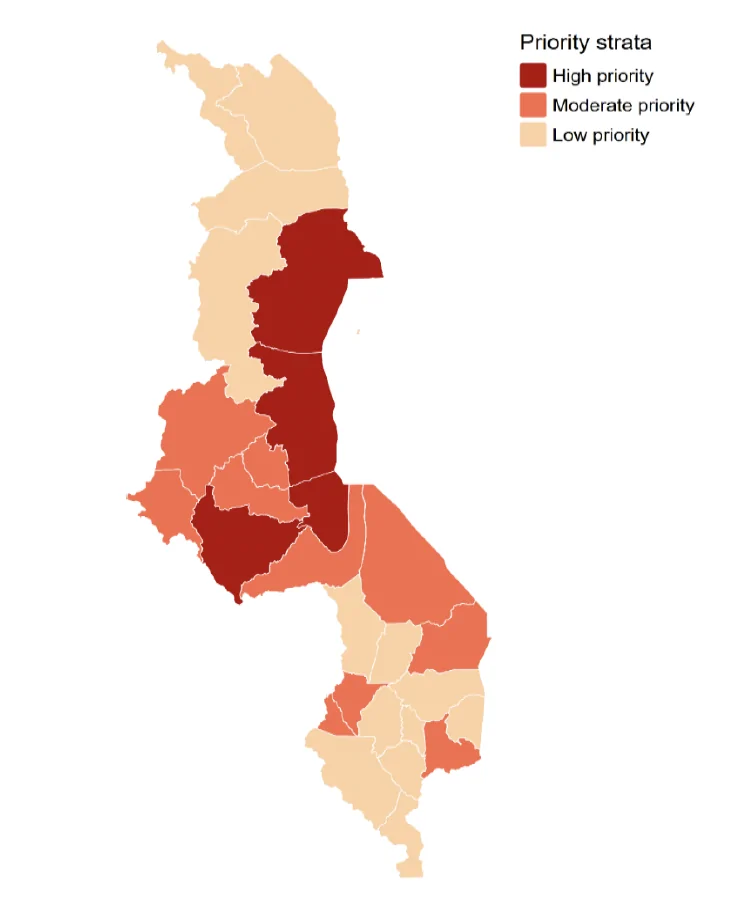
Spatial distribution of malaria chemoprevention priority strata across districts in Malawi.

### Non-spatial panel model results

Table 4 presents the results from fixed effects panel models with district-level clustered standard errors. Rainfall shows a positive and statistically significant association with most malaria outcomes, including total cases, under-five cases, and incidence rates. Rainfall anomalies are negatively associated with malaria outcomes. Temperature variables show weaker and mostly insignificant effects. Population size is negatively associated with several malaria indicators.

**Table 4:** Non-spatial fixed effects panel model results for malaria transmission determinants. Note: Fixed effects models with district-level clustering. Coefficients with standard errors in parentheses. Significance: *** p &lt; 0.001, ** p &lt; 0.01, * p &lt; 0.05.

Variable	Total cases	Under-5 cases	Incidence all ages	Incidence under-5	Total deaths
Rainfall	22.5953*** (3.3267)	4.7712*** (0.9456)	0.0344*** (0.0051)	0.036*** (0.0073)	0.0031 (0.0016)
rain_anomaly	-25.6082*** (6.6442)	-5.0939** (1.8887)	-0.0463*** (0.0102)	-0.0457** (0.0146)	-0.0047 (0.0033)
mean_temp	-125.7622 (190.6972)	-62.4382 (54.2068)	-0.0877 (0.2923)	-0.3717 (0.42)	-0.1541 (0.0937)
max_temp	-247.3317 (139.5183)	-42.9717 (39.6589)	-0.4553* (0.2138)	-0.4267 (0.3073)	-0.0248 (0.0686)
min_temp	49.4544 (81.4262)	16.8714 (23.1459)	0.0316 (0.1248)	0.0984 (0.1793)	0.0844* (0.04)
pop_all	-0.0617*** (0.0181)	-0.0336*** (0.0051)	0 (0)	-2e-04*** (0)	-1e-04*** (0)

To account for spatial dependence in malaria transmission across districts, spatial econometric models were estimated. These models allow malaria outcomes in one district to be influenced by conditions in neighbouring districts. Two spatial specifications were considered: the Spatial Autoregressive (SAR) model, which captures spatial spillover effects in the dependent variable, and the Spatial Error Model (SEM), which accounts for spatial correlation in unobserved factors.

Table 5 presents the results from the spatial autoregressive SAR model. Rainfall remains positively associated with malaria outcomes, particularly total cases and incidence measures. Rainfall anomalies show a negative association with malaria incidence. The spatial autoregressive parameter (*λ*) is positive and statistically significant across all specifications, indicating strong spatial dependence in malaria outcomes across neighbouring districts.

**Table 5:** Spatial Autoregressive (SAR) model results for determinants of malaria transmission. Note: Covariates: Rainfall, rain_anomaly, mean_temp, max_temp, min_temp, pop_all. Coefficients with standard errors in parentheses. Significance: *** p &lt; 0.001, ** p &lt; 0.01, * p &lt; 0.05.

Variable	Total cases	Under-5 cases	Incidence all	Incidence under-5	Total deaths
Rainfall	10.178*** (2.907)	1.799* (0.805)	0.022*** (0.005)	0.018** (0.006)	0.002 (0.002)
rain_anomaly	-10.749 (5.748)	-1.395 (1.601)	-0.030** (0.009)	-0.020 (0.013)	-0.003 (0.003)
mean_temp	-94.919 (164.766)	-46.372 (46.020)	-0.026 (0.270)	-0.166 (0.369)	-0.122 (0.091)
max_temp	-140.557 (120.205)	-18.388 (33.566)	-0.374 (0.197)	-0.362 (0.269)	-0.025 (0.066)
min_temp	46.458 (70.186)	16.260 (19.601)	0.051 (0.115)	0.136 (0.157)	0.072 (0.039)
pop_all	-0.046** (0.016)	-0.025*** (0.004)	-0.000 (0.000)	-0.000* (0.000)	-0.000*** (0.000)
λ	0.459*** (0.030)	0.487*** (0.029)	0.340*** (0.034)	0.437*** (0.031)	0.208*** (0.037)

Table 6 reports the results from the SEM. Rainfall continues to show a positive association with malaria outcomes, while rainfall anomalies maintain a negative relationship with malaria incidence. The spatial error parameter (*ρ*) is positive and statistically significant in most models, confirming the presence of spatially correlated unobserved factors influencing malaria transmission across districts.

**Table 6:** Spatial Error Model (SEM) results for determinants of malaria transmission. Note: Covariates: Rainfall, rain_anomaly, mean_temp, max_temp, min_temp, pop_all Coefficients with standard errors in parentheses. Significance: *** p &lt; 0.001, ** p &lt; 0.01, * p &lt; 0.05.

Variable	Total cases	Under-5 cases	Total deaths	Death incidence
Rainfall	14.951** (4.749)	2.552 (1.392)	0.003 (0.002)	0.000 (0.000)
rain_anomaly	-15.807* (7.322)	-2.330 (2.083)	-0.004 (0.003)	-0.000 (0.000)
mean_temp	-425.510* (191.028)	-138.691* (53.958)	-0.144 (0.096)	-0.000 (0.000)
max_temp	-45.733 (127.946)	6.270 (35.490)	-0.025 (0.069)	-0.000 (0.000)
min_temp	89.147 (73.727)	29.497 (20.452)	0.068 (0.040)	0.000 (0.000)
pop_all	-0.074*** (0.020)	-0.039*** (0.006)	-0.000*** (0.000)	-0.000** (0.000)
ρ	0.471*** (0.030)	0.511*** (0.028)	0.204*** (0.038)	0.075 (0.040)

## Discussion

This study investigated the spatial, climatic and population determinants of malaria transmission in Malawi using district-level panel data integrated with spatial econometric modelling to identify areas that could benefit most from targeted malaria chemoprevention. Four principal findings emerged. First, malaria transmission remains highly heterogeneous, with persistent hotspots concentrated in the southern region and lakeshore districts. Second, rainfall was the strongest and most consistent climatic determinant of malaria transmission, whereas temperature exhibited more complex, non-linear effects. Third, significant spatial dependence indicates that malaria transmission extends beyond administrative boundaries, highlighting the importance of accounting for geographical interactions when modelling malaria risk. Finally, the composite chemoprevention priority index successfully identified districts where high malaria burden, favourable climatic conditions, and large exposed populations converge, providing an evidence-based framework for geographically targeted malaria prevention.

These findings are broadly consistent with the Ross–Macdonald framework, which describes malaria transmission as the interaction between mosquito vectors, human hosts, and environmental conditions. Rainfall emerged as the dominant climatic predictor across all malaria outcomes, supporting its established role in creating and sustaining mosquito breeding habitats and increasing vector survival. Conversely, rainfall anomalies were associated with lower malaria burden, suggesting that departures from normal rainfall patterns may disrupt breeding habitats through flooding or prolonged dry periods. Temperature demonstrated the expected non-linear relationship with malaria transmission: higher minimum temperatures were associated with increased malaria burden, whereas higher maximum temperatures were associated with reduced transmission, consistent with evidence that temperatures above the optimal range reduce mosquito survival and parasite development [41,42]. Collectively, these findings reinforce the importance of climatic suitability as a major ecological driver of malaria transmission in Malawi.

An important finding of this study is that the timing of peak malaria transmission differs considerably across districts. Although most districts experienced peak transmission between March and July, others had earlier or later transmission periods extending across different parts of the year. These differences may reflect variation in rainfall, temperature, altitude, and local ecological conditions that influence mosquito breeding and parasite development. The observed temporal heterogeneity suggests that malaria interventions should be aligned with local transmission patterns rather than implemented according to a single national schedule. This is particularly relevant for time-sensitive interventions such as seasonal malaria chemoprevention, indoor residual spraying, and intensified surveillance, where implementation before or at the beginning of the peak transmission period is important for maximising impact [42,43].

Marked spatial heterogeneity was evident across the country, with districts in southern Malawi and along the lakeshore consistently experiencing the highest malaria burden. These findings are consistent with previous national mapping studies showing that these areas continue to sustain intense malaria transmission despite substantial investments in malaria control [9,12,44]. Furthermore, the significant spatial autoregressive and spatial error parameters demonstrate that malaria transmission is influenced not only by local environmental conditions but also by processes operating across neighbouring districts. Shared ecological conditions, population mobility, and cross-district transmission are likely contributors to these geographical spillover effects. Consequently, conventional regression models that ignore spatial dependence may underestimate transmission dynamics and produce less reliable estimates, highlighting the value of incorporating spatial econometric methods into malaria surveillance and programme planning.

An unexpected finding was the consistently negative association between population size and several malaria outcomes. Because the fixed-effects models estimate changes within districts over time rather than differences between districts, this result should not be interpreted as evidence that more populous districts experience lower malaria burden. Instead, it may reflect concurrent changes associated with urbanisation, housing, healthcare access, or malaria control interventions that were not explicitly measured in this study. However, the relationship between urbanisation and malaria is complex, and recent evidence shows that transmission may persist in densely populated urban settings, particularly where urban growth is unplanned or population mobility is high [45]. Similarly, malaria mortality exhibited weaker spatial dependence than malaria incidence. This may reflect the fact that deaths depend not only on transmission intensity but also on timely diagnosis, treatment availability, transportation, healthcare accessibility, and the quality of care. Recent evidence shows that limited transport and healthcare access contribute to delays in malaria treatment, increasing the risk of severe disease and death [46]. Routine mortality data may also be affected by underreporting and misclassification, which can reduce the precision of spatial estimates [1,42]. These findings should therefore be interpreted cautiously and warrant further investigation using more detailed health-system and mortality data.

A major contribution of our study is the development of a composite chemoprevention priority index that integrates malaria burden, climatic suitability, and population exposure to identify districts most likely to benefit from malaria chemoprevention. Unlike approaches based solely on malaria incidence, this multidimensional framework captures both epidemiological need and environmental suitability, providing a stronger basis for strategic decision-making. The close agreement between the priority index and the observed spatial distribution of malaria burden further supports its validity. These findings are consistent with evidence demonstrating that malaria chemoprevention achieves the greatest public health impact when implemented in geographically appropriate settings [1,5,42]. Although Malawi differs from the highly seasonal Sahelian countries where Seasonal Malaria Chemoprevention is routinely implemented, the substantial spatial heterogeneity observed suggests that geographically targeted prevention strategies could improve programme efficiency compared with a uniform national approach.

The findings have important implications for malaria control in Malawi. Persistent transmission hotspots indicate that intervention planning should increasingly account for geographical heterogeneity when prioritising resources and selecting implementation areas. The observed variation in malaria seasonality across districts further suggests that interventions should be scheduled to coincide with local transmission patterns rather than follow a single national implementation calendar. Delivering interventions within the peak transmission is likely to maximise their effectiveness. In addition, the strong association between rainfall and malaria transmission indicates that seasonal climate information could be used to guide the timing of preventive interventions, strengthen surveillance preparedness, and improve resource allocation during periods of increased malaria risk. These approaches are consistent with the World Health Organization's recommendations for stratified malaria control. However, this study was designed to identify priority areas rather than determine the operational implementation of malaria chemoprevention. Decisions regarding the choice of intervention, target population, implementation schedule, and programme costs will require additional demographic, operational, and health-system data beyond the scope of the present study.

This study has several strengths. It combined district-level surveillance, climatic, and population data with spatial econometric modelling to account for geographical dependence and developed a multidimensional framework for identifying priority districts for malaria chemoprevention. Nevertheless, several limitations should be acknowledged. The analysis relied on routinely collected surveillance data, which may be affected by reporting inaccuracies, particularly for mortality. In addition, longitudinal district-level data on healthcare access, intervention coverage, and socioeconomic conditions were not consistently available and therefore could not be included in the models. Future studies should integrate these factors and evaluate the operational feasibility and cost-effectiveness of implementing targeted malaria chemoprevention in the priority districts identified by this framework.

## Conclusions

This study showed that malaria transmission in Malawi varies considerably across districts and is strongly influenced by climatic conditions and spatial processes. The findings identified districts where malaria chemoprevention is likely to have the greatest impact and highlight the importance of considering geographical differences when planning malaria control interventions. The prioritisation approach presented in this study provides a basis for supporting malaria programme planning and may help inform the future implementation of malaria chemoprevention in Malawi.
